# Qat Chewing and Periodontal Pathogens in Health and Disease: Further Evidence for a Prebiotic-Like Effect

**DOI:** 10.1155/2015/291305

**Published:** 2015-08-16

**Authors:** Abdulrahman Al-Alimi, Tara Taiyeb-Ali, Nasruddin Jaafar, Nezar Noor Al-hebshi

**Affiliations:** ^1^Department of Oral Biology & Biomedical Sciences (Periodontology), Faculty of Dentistry, University of Malaya, 50603 Kuala Lumpur, Malaysia; ^2^Department of Community Oral Health & Clinical Prevention, Faculty of Dentistry, University of Malaya, 50603 Kuala Lumpur, Malaysia; ^3^Department of Preventive Dentistry-Periodontology, Faculty of Dentistry, Jazan University, Jazan 45142, Saudi Arabia

## Abstract

*Aim*. Qat chewing has been reported to induce subgingival microbial shifts suggestive of prebiotic-like properties. The objective here was to assess the effect of qat chewing on a panel of classical and new putative periopathogens in health and periodontitis. *Materials and Methods*. 40 qat chewers and 40 nonchewers, equally stratified by periodontal health status, were recruited. Taqman, real-time PCR was used to quantify total bacteria, *Porphyromonas gingivalis*, *Tannerella forsythia*, *Treponema denticola*, *Parvimonas micra*, *Filifactor alocis*, Synergistetes, and TM7s in pooled subgingival biofilm samples. Differences in microbial parameters between the study groups were analysed using ordinal regression. *Results*. In health, the qat chewers harboured significantly lower relative counts of *P. gingivalis*, *T. forsythia*, Synergistetes, and TM7s after adjustment for multiple comparisons (*P* ≤ 0.007). At nominal significance level, they also carried lower counts of TM7s and *P. micra* (*P* ≤ 0.05). In periodontitis, the chewers had lower counts of all taxa; however, only *T. denticola* withstood correction for multiple comparisons (*P* ≤ 0.0063). *Conclusions*. Qat chewing is associated with lower proportions of periopathogens, particularly in subjects with healthy periodontium, which supports previous reports of its prebiotic-like properties. This potentially beneficial biological effect can be exploited by attempting to isolate the active fraction.

## 1. Introduction

Qat, commonly spelled as khat in the literature, is the common name for* Catha edulis*, an evergreen plant of the family Celastraceae that is widely cultivated on the hill and mountain sides of Yemen and Ethiopia [1]. Millions of people in these and neighboring countries habitually chew the fresh leaves and twigs of this shrub for their stimulating, amphetamine-like effects that are attributed to the active amines cathinone and cathine [2]. The habit is several centuries old and has been confined to and around geographical areas where the plant is cultivated. Recently, however, the habit has spread worldwide with immigrants, despite its prohibition by many countries. Typically, 100–200 grams of fresh plant material are chewed into a bolus that is retained against the cheek on one side of the mouth. The chewing session usually lasts for several hours and takes the form of a social gathering [1]. Detailed information about the different aspects of qat including its chemistry, pharmacology, and medical and dental effects can be found in the literature [1, 3–6].

Apart from being a substance of abuse and the controversy about the social and medical adverse effects associated with its long-term use, qat seems to have some potential as a medicinal plant. According to Arabic sources that date back to the 11th century, qat was used as a medicinal plant in the region of Turkistan and Afghanistan [7]. In Ethiopia, the processed leaves and roots of qat are traditionally prescribed for treating various chest problems [8]. Administered to experimental animals, qat has been shown to lower serum glucose, triglycerides, and cholesterol levels [9]. In 1999, Elhag et al. [10] isolated two compounds from qat callus with potent antibacterial activity against some bacteria including* Bacillus subtilis*,* Staphylococcus aureus*, and* Mycobacterium *ssp. and strong cytotoxic effect against prostate and leukemia cell lines. Later work elucidated unique molecular mechanisms by which qat induces apoptosis in leukemia cells [11–13], proposing it as a potential source of novel therapeutics. In another example, two metabolites of cathinone, cathine and norephedrine, have been found to boost sperms fertilizing ability* in vitro* [14].

There is also some evidence to suggest that qat chewing positively influences the oral microbial ecology. In the first study to compare the microbial profile of dental biofilm from qat chewers and nonchewers using checkerboard DNA-DNA hybridization, qat chewing was found to be associated with higher levels of some health-compatible periodontal bacteria such as* Veillonella parvula* and lower levels of the periodontal pathogen* Tannerella forsythia *[15]. In a later* in vitro* study, aqueous crude extracts of qat were demonstrated to possess selective antibacterial activity against periodontal bacteria, with the highest activity against pathogenic species, including* Porphyromonas gingivalis* and* T. forsythia,* and least activity against health-compatible ones [16]. Tested in the Zurich biofilm model, the qat extracts resulted in microbial composition shifts characterized by dramatic increase in proportions of* Streptococcus oralis* paralleled with a drop in total anaerobes, a profile that is compatible with periodontal health [17]. In line with this, a more recent study employing real-time PCR showed that subgingival biofilm from qat chewers with chronic periodontitis harbored lower proportions of* P. gingivalis*,* Parvimonas micra*, prevotellae, and fusobacteria in both healthy and diseased sites [18].

So far, studies have assessed the effect of qat chewing on the levels of classical pathogens, that is, members of the red and orange complex [19]; however, its effect on new putative pathogens, such as* Filifactor alocis* and oral phylotypes of phyla Synergistetes and TM7 (referred to hereafter as oral Synergistetes and oral TM7s, resp.), has not been investigated yet. The objective of this study was, therefore, to reexamine the effect of qat chewing on classical periodontal pathogens using a larger sample size and to assess, for the first time, its effect on new putative pathogens.

## 2. Materials and Methods

### 2.1. Study Design and Subjects

This was a cross-sectional, comparative study with parallel-arm design. Eighty, 30–60-year-old study subjects were recruited from among outpatients attending Al-Thawra General Hospital and a private dental centre in Sana'a City between February and July 2013. The study subjects were selected based on their periodontal health status and qat chewing history as follows: qat chewers with healthy periodontium (*n* = 20), qat nonchewers with healthy periodontium (*n* = 20), qat chewers with chronic periodontitis (*n* = 20), and qat nonchewers with chronic periodontitis (*n* = 20). Clinically, subjects were examined using the community periodontal index (CPI) and clinical attachment loss (CAL) according to WHO [21]. Plaque index [22] was recorded on index teeth. All measurements were made by single examiner (Al-Alimi A). A subject was classified as having chronic periodontitis if presented with at least one index tooth per quadrant with pocket depth of ≥5 mm (CPI score ≥ 3; 3.5–5.5 band on the WHO probe invisible). Having no CPI score >2 was used to define a subject with healthy periodontium. A history of qat chewing for 5 or more years at a frequency of at least 1 day per week and a minimum chewing session duration of 3 hours was used to define a qat chewer. History about other habits including cigarette and water pipe smoking was obtained. Subjects with less than 20 remaining teeth or history of periodontal treatment, antibiotic intake in the last three months, or any condition/disease known to modify subgingival microbial composition were excluded. Ethical approval to carry out the study was obtained from the Faculty of Dentistry, Sana'a University; all study subjects gave written consent to participate in the study.

### 2.2. Sample Collection and DNA Extraction

Subgingival biofilm samples were collected from the participants using sterile paper points (size 40; Megadenta, Germany). In the subjects with chronic periodontitis, the deepest pocket in each quadrant was sampled, while in those with healthy periodontium, samples were obtained from one site per quadrant. The paper point samples from each subject were pooled in TE buffer and stored at −20°C.

At the time of DNA extraction, the samples were thawed and centrifuged at 15,000 g for 1 min to pellet bacterial cells. DNA was extracted from the resultant cell pellets using the Purelink Genomic DNA extraction kit (Life Technologies, USA), according to the manufacturer's protocol for Gram positive bacteria. DNA from each sample was eluted in a final volume of 100 *μ*L and stored at −80°C.

### 2.3. Quantitative PCR Assays

Previously validated Taqman q-PCR assays were used to quantify total bacteria, 4 classical periodontal pathogens, and three recently suspected pathogenic taxa in the DNA extracts (Table 1). The primers/probe sets (sequences shown in Table 1) were obtained from PrimerDesign, a UK-based company, as optimized, ready to use kits that also included plasmid-based, positive controls for construction of quantification standard curves. The q-PCR reaction setup and amplification were carried out as previously described [20]. Absolute counts of the test species/phylotypes were determined in DNA copies/sample; these were then normalized to total bacterial counts to obtain relative counts (% total bacteria). The assays were previously demonstrated to have high specificity and efficiency and a detection limit of 100–200 DNA copies/sample.

### 2.4. Statistical Analysis

Clinical and microbiological data were described as percentages or medians with interquartile ranges as appropriate. Significance of differences between the qat chewers and nonchewers in clinical variables was sought using Chi squared test for categorical variables and Mann-Whitney test for scale variables. Differences in absolute counts (log-transformed) and relative counts of the test taxa between the chewers and nonchewers, for the chronic periodontitis and healthy periodontium groups separately, were tested using multiple ordinal regression, including demographic variables, other oral habits, and mean plaque index as covariates. The complementary log-log and negative-negative log functions were used for absolute and relative counts, respectively. Two levels of significance were employed: a nominal *P* value of 0.05 and Bonferroni-adjusted *P* values of 0.0063 and 0.007 for absolute and relative counts, respectively (correction for multiple comparisons). All statistical analyses were performed using SPSS version 20 (IBM, USA).

## 3. Results

### 3.1. Clinical Characteristics

The clinical features of the study groups are shown in Table 2. In the subjects with healthy periodontium, the qat chewers had significantly higher median PI and CAL scores compared to the nonchewers. No significant differences in periodontal parameters between the qat chewers and nonchewers with chronic periodontitis were observed; however, the qat chewers included significantly more males and cigarette smokers. A similar trend was seen in the subjects with healthy periodontium but the differences were not significant. Overall, the chewers reported habitual use of qat for an average of 15.4 years (range of 5–30 years) at an average frequency of 5 days a week (range of 2–7 days) and an average session duration of 5 hours (range of 3–12 hours).

### 3.2. Qat Chewing and Microbial Counts in Health

**Figure 1:**
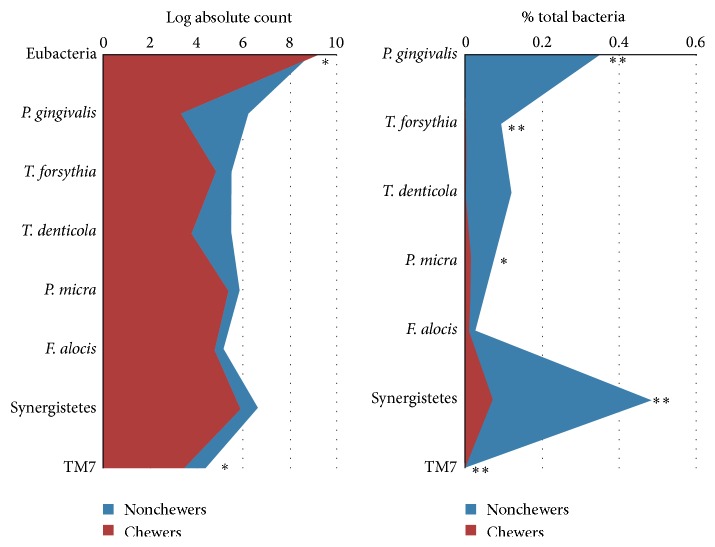
Median absolute and relative counts of the test taxa in subgingival biofilm from the qat chewers and nonchewers with healthy periodontium. Significance of difference was sought using ordinal regression adjusting for demographic variables, other oral habits, and mean plaque index.  ^*∗*^Significant at 0.05.  ^*∗∗*^Significant after correcting for multiple comparisons (*P* < 0.007).

The log-transformed absolute counts and relative counts (proportions) of the test taxa in the qat chewers and nonchewers with healthy periodontium are shown in Figure 1. Although the qat chewers had significantly higher total subgingival bacterial load (*P* ≤ 0.05), they harbored lower absolute and relative counts of all the test species/phylotypes than did the nonchewers. The differences were, however, mostly significant for relative counts. At the adjusted significance level (*P* ≤ 0.007), qat chewing was associated with lower relative counts of* P. gingivalis*,* T. forsythia*, oral Synergistetes, and TM7s. At nominal significance level (*P* ≤ 0.05), it was also associated with lower relative counts of* P. micra. *In contrast, the differences in absolute counts reached nominal significance (*P* ≤ 0.05) only for oral TM7s.

### 3.3. Qat Chewing and Microbial Counts in Periodontitis

**Figure 2:**
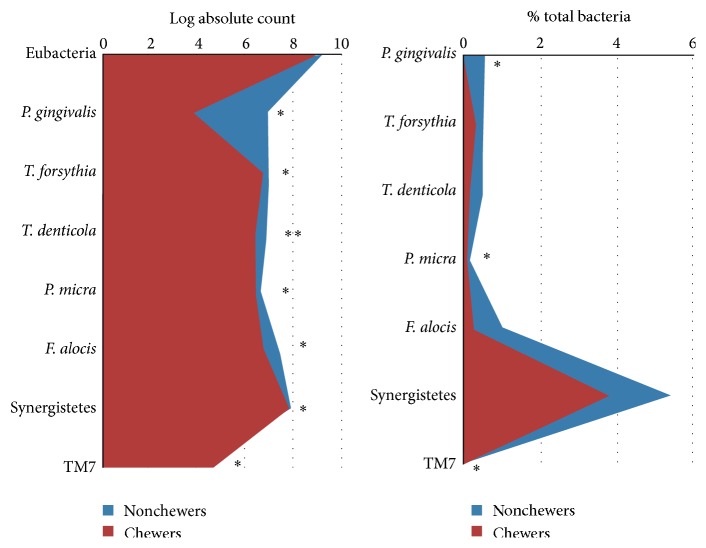
Median absolute and relative counts of the test taxa in subgingival biofilm from the qat chewers and nonchewers with chronic periodontitis. Significance of difference was sought using ordinal regression adjusting for demographic variables, other oral habits, and mean plaque index.  ^*∗*^Significant at 0.05.  ^*∗∗*^Significant after correcting for multiple comparisons (*P* < 0.0063).

Figure 2 illustrates the differences in absolute and relative counts of the test taxa between the qat chewers and nonchewers with chronic periodontitis. At nominal significance level (*P* ≤ 0.05), all the test species/phylotypes, except TM7s, were present at lower absolute counts in the qat chewers; however, only* T. denticola* maintained significant difference after adjustment for multiple comparisons (*P* ≤ 0.0063). The relative counts of all the test taxa, again except for TM7s, were also lower in the qat chewers, but the differences reached nominal significance (*P* ≤ 0.05) only for* P. gingivalis* and* P. micra*; none withstood correction for multiple comparisons. The differences in absolute and relative counts of TM7s were nominally significant but in the opposite direction; that is, they were higher in the qat chewers.

## 4. Discussion

Qat is a recreational drug with a potential of abuse. However, compared to other 19 drugs of abuse, qat was found to be among the least addictive and to be associated with the least physical and social harm [23]. Nevertheless, the habit has been linked in the literature to several adverse medical health problems including cardiovascular events, acute liver toxicity, and mental health problems especially among heavy, long-term users [4, 6, 24]. It has also been reported to have detrimental effects on oral hard and soft tissues, although with some controversy [1]. These include increased periodontal attachment loss [25], mucosal changes [26], and temporomandibular disorders [27]. On the other hand, however, qat seems to have some potentially beneficial biological effects (see Section 1) that can be exploited in a context other than its traditional use as a drug and should, therefore, not be ignored.

The current study is a continuation of previous work aimed at elucidating the effect of qat on ecology of subgingival dental biofilm [15, 16, 18]. The test panel included 4 classical periodontal pathogens (red complex +* P. micra*) for reassessment and, for the first time, 3 of the newly implicated putative pathogenic species/phylotypes (*F. alocis*, oral Synergistetes, and oral TM7s). To make it more comprehensive than previous studies, the effect of qat on the test panel was assessed in both subjects with healthy periodontium and those with chronic periodontitis. The subgingival biofilm DNA samples of the study subjects were analyzed using Taqman q-PCR which, in addition to being very sensitive and specific, allows for relative quantification of target taxa by normalizing their absolute counts to total bacterial counts. The study, however, has its limitations. First, the sample size was decided based on feasibility rather than formal calculations; the limited sample size may, therefore, compromise generalizability of the results. Second, the study could have been designed to allow comparison between the chewing and nonchewing sides (split mouth design) in addition to that between the chewers and nonchewers (parallel-arm design), which was not done. Finally, the study missed to include health-compatible taxa in the test panel, for example, streptococci, which would have allowed more reliable assessment of the prebiotic activity of qat.

For the sake of the discussion below, more importance is given to differences in relative counts rather than absolute counts for two reasons. Firstly, relative quantification adjusts for variations in the amounts of samples collected and input DNA and is thus more reliable for comparisons among samples than absolute counts [28, 29]. Secondly, chronic periodontitis is increasingly recognized as an ecological disease that is associated with shifts in the microbial composition of subgingival dental biofilm and thus changes in proportions (relative counts) of the different species within the microbial community [30].

Regardless of statistical significance, the qat chewers with healthy periodontium harbored lower relative counts of all tested taxa compared to the nonchewers in the same group. However, the differences were particularly evident for* P. gingivalis*,* T. forsythia*, Synergistetes, and TM7, withstanding adjustment for multiple comparisons. For the former two species, the findings substantiate those from previous studies on samples from periodontally intact subjects or sites [15, 18]. They are also perfectly consistent with a previous* in vitro* study, in which both species were found to be among the most sensitive periodontal bacteria tested to aqueous crude qat extracts [16]. It is important to realize that* P. gingivalis* and* T. forsythia* are members of the red complex [19]. In addition, oral Synergistetes, whose counts are shown here to negatively correlate with qat chewing, have been very recently suggested to represent an additional member of the red complex [20]. Together, these findings suggest that qat chewing selectively suppresses growth of the red complex in subgingival biofilm. In fact, the qat chewers also tended to harbor lower relative counts of* T. denticola* (the third classical member of the red complex), which despite being not statistically significant is in harmony with the conception above and, most importantly, undermines findings from a previous study in which qat chewing was found to be associated with higher counts of this important periopathogen [18].

In the periodontitis group, the differences in relative counts reached nominal significance only for* P. gingivalis *and* P. micra*, while TM7s were even detected at higher counts in association with the habit. Like* P. gingivalis*,* P. micra* has also been previously shown to be present in significantly lower counts in periodontal pockets of qat chewers [18] and to be sensitive to qat extracts* in vitro *[16]. As in the periodontally healthy group,* T. denticola *also tended to be present in lower relative counts in association with qat chewing, which is again in contradiction with the previous report [18]. Overall, the effect of qat chewing was much less pronounced in the periodontitis group. This, however, probably represents a diffusion limitation; that is, the active compounds of qat probably do not reach the concentrations required to exert an effect on microbiota in deep pockets. In fact, cigarette smoking has been previously shown to induce subgingival microbial shifts only in pockets ≤4 mm in depth [31].

Significantly more total bacterial DNA was recovered from the qat chewers in the periodontally healthy group, suggesting that while qat suppresses growth of periodontal pathogens in subgingival biofilm it promotes growth of other species, probably the beneficial ones as may be extrapolated from previous studies. For example, qat extracts have been shown to dramatically increase the proportions of* S. oralis*, in the Zurich biofilm at the expense of the anaerobic species [17]. In addition, qat chewers have also been shown to harbor higher counts of* V. parvula*, another health-compatible species [15]. It can, therefore, be hypothesized that qat lowers the proportions of periodontal pathogens not only by selectively inhibiting them but also by promoting growth of beneficial species that, in turn, act as probiotics to restrict growth of the pathogens. As such, the effects of qat on periodontal microbiota can be described as being prebiotic-like. A prebiotic has been recently defined as “a selectively fermented ingredient that allows specific changes, both in the composition and/or activity in the gastrointestinal microflora that confers benefits upon host well-being and health” [32].

As the definition indicates, the concept of prebiotics has evolved and has been extensively investigated almost exclusively in connection with gastrointestinal health. With the paradigm shift towards a microbial community-based understanding of oral diseases, there seems to be an increasing interest in the possibility of applying prebiotics to oral health [33]. However, there have been hardly any attempts to address the utility of this approach or to explore for foods or natural products that can manipulate the composition of oral microflora in favor of oral health. In fact, qat may be the only substance so far to be described as having prebiotic-like properties in connection with oral microbiota. Unfortunately, these findings seem to have attracted little attention, probably because qat is a drug of abuse, although they do provide enough evidence to whet dental researchers' appetite for pursuing similar research using other foods or natural products. As far as qat is concerned, there is probably now enough evidence to justify making an attempt to isolate/identify the active compound(s) responsible for its prebiotic-like properties. It is also important to assess its effect on broader number of oral bacteria including beneficial species, to confirm whether it strictly fulfills the classical definition of a prebiotic.

In conclusion, qat chewing is shown here to be associated with lower proportions of periodontal pathogens belonging to the red complex, particularly in the subjects with healthy periodontium, which supports previous claims on its prebiotic-like effects on subgingival periodontal microbiota. The findings are not meant to advocate qat use by the public but to highlight the possibility of applying the prebiotic concept to oral health and to stimulate more research on this area, including isolation of potentially active compounds from qat.

## Figures and Tables

**Table 1:** Sequences of the primers and probes used in the quantitative PCR assays [20].

Test species	Sequences 5′-3′	Target gene	Product size
Total bacteria	F-primer: AAACTCAAAGGAATTGACGGGG R-primer: TTGCGCTCGTTGCGGGACT Probe: FAM-CTGTCGTCAGCTCGTGTCGTGA-BHQ	16S rRNA	205 bp

*P*. *gingivalis*	F-primer: ACGAATCAAAGGTGGCTAAGTT R-primer: TTAGTCGCATTTTCGGCTGAT Probe: FAM-CCTGCTGTTCTCCATTATAAACCATTACGG-BHQ	fimA	85 bp

*T*. *forsythia*	F-primer: GATAGGCTTAACACATGCAAGTC R-primer: GTTGCGGGCAGGTTACATAC Probe: FAM-TTACTCACCCGTGCGCCGGTCG-BHQ	16S rRNA	99 bp

*T*. *denticola*	F-primer: GGGCGGCTTGAAATAATRATG R-primer: CTCCCTTACCGTTCGACTTG Probe: FAM-CAGCGTTCGTTCTGAGCCAGGATCA-BHQ	16S rRNA	92 bp

*P*. *micra*	F-primer: TGAGCAACCTACCTTACACAG R-primer: GCCCTTCTTACACCGATAAATC Probe: FAM-ACCGCATGAGACCACAGAATCGCA-BHQ	16S rRNA	112 bp

Oral Synergistetes^¶^	F-primer: GGAGTACGGTCGCAAGATTG R-primer: GTAAGGTTCTTCGGTTTGCATC Probe: FAM-ACAAGCGGTGGAGCACGTGGTTTAAT-BHQ	16S rRNA	98 bp

*Filifactor alocis*	F-primer: ACCCTCAAGTTGCCAAAATTATTAT R-primer: TACTCCCTTTCTTCTGGTTAAATCT Probe: FAM-TCGCTCTTTTTGCCGCCTCTCTTGC-BHQ	16S rRNA	101 bp

Oral TM7s^§^	F-primer: GCTCGTGTCGTGAGATGTTT R-primer: ATCCCCTCCTTCCTCCCCG Probe: FAM-TAAGTCCATCAACGAGCGCAACCCTT-BHQ	16S rRNA	107 bp

^¶^The primers/probe set covers *Fretibacterium fastidiosum*, *Fretibacterium* sp. oral taxons 358, 359, 360, 361, 362, 452, and 453, *Jonquetella anthropi*, and *Pyramidobacter piscolens*.

^§^The primers/probe set covers TM7 oral taxons 347, 348, 350, 351, 355, 356 (I025), and 437.

**Table 2:** Clinical characteristics of the qat chewers and qat nonchewers by periodontal health status.

Variable	Health		*P* ^¶^	Periodontitis		*P* ^¶^
	Qat nonchewers	Qat chewers		Qat nonchewers	Qat chewers	
	*n* = 20	*n* = 20		*n* = 20	*n* = 20	
Age, median (interquartile range)	32.0 (31.3–33.8)	32.0 (31.0–33.8)	NS	44.0 (36.3–50.0)	40.0 (32.5–45.0)	NS
% males	60%	80%	NS	70%	95%	0.05
% cigarette smokers	20%	35%	NS	35%	80%	0.005
% water pipe smokers	0%	10%	NS	15%	20%	NS
Plaque index, median (interquartile range)	0.17 (0.13–0.25)	0.41 (0.24–1.03)	0.001	1.67 (1.44–1.96)	1.73 (1.59–1.92)	NS
Mean CPI score, median (interquartile range)^*∗*^	0.00 (0.00-0.00)	0.00 (0.00–0.17)	NS	2.67 (2.50–3.00)	2.58 (2.38–2.83)	NS
Mean CAL score, median (interquartile range)^*∗*^	0.00 (0.00-0.00)	0.00 (0.17–0.29)	0.004	0.83 (0.54–1.63)	1.00 (0.54–2.00)	NS

^¶^ *P* value: Chi square for categorical variables and Mann-Whitney test for scale variables. NS: not significant.

^*∗*^Mean CPI/CAL score was calculated for each study subject (average of six scores). At the group level, the data (mean CPI/CAL scores) were presented as medians and interquartile ranges.
